# Assessment of Anti-*Toxoplasma gondii* Antibodies in Patients with Chronic Hepatitis C Virus Infection: Evaluation of Liver Fibrosis Biomarkers and Elastography Findings

**DOI:** 10.3390/tropicalmed11100293

**Published:** 2026-10-16

**Authors:** Mona Mohamed Abdulwehab, Enas A. El Saftawy, Shaimaa Elattar, Ahmed M. Abdel Hamied, Sara O. Elgendy, Alshaimaa M. M. Eid, Asmaa Saleh, Raneem Alzeer, Mohammed Gamal, Ragaey Ahmad Eid, Amal M. Mahfoz, Hanaa O. Fadl

**Affiliations:** 1 Clinical Pathology Department, Faculty of Medicine for Girls, Al-Azhar University, Cairo 11651, Egypt; monabdulwehab86@gmail.com (M.M.A.); shymaa.mohamed12@azhar.edu.eg (S.E.); 2 Medical Parasitology Department, Faculty of Medicine, Cairo University, Cairo 11562, Egypt; enas.ali.omar@kasralainy.edu.eg (E.A.E.S.); hoabdelmohsen@kasralainy.edu.eg (H.O.F.); 3 Medical Parasitology Department, Faculty of Medicine, Armed Forces College of Medicine, Cairo 11774, Egypt; 4 Department of Pediatrics, Faculty of Medicine, Nahda University (NUB), Beni-Suef 19206, Egypt; ahmed.abdelhamid@nub.edu.eg; 5 Clinical and Chemical Pathology Department, Faculty of Medicine, Beni-Suef University, Beni-Suef 62521, Egypt; 6 Department of Hepatology, Gastroenterology and Infectious Diseases, Faculty of Medicine for Girls, Al-Azhar University, Cairo 11754, Egypt; drshimaa610@gmail.com; 7 Department of Pharmaceutical Sciences, College of Pharmacy, Princess Nourah bint Abdulrahman University, P.O. Box 84428, Riyadh 11671, Saudi Arabia; asali@pnu.edu.sa; 8 Department of Pharmaceutical Practice, College of Pharmacy, Princess Nourah bint Abdulrahman University, P.O. Box 84428, Riyadh 11671, Saudi Arabia; raalzeer@pnu.edu.sa; 9 Department of Pharmaceutical Analytical Chemistry, Faculty of Pharmacy, Beni-Suef University, Alshaheed Shehata Ahmed Hegazy St., Beni-Suef 62574, Egypt; mgamalm3000@yahoo.com; 10 Department of Gastroenterology, Hepatology and Infectious Diseases (Tropical Medicine Department), Faculty of Medicine, Beni-Suef University, Beni-Suef 62521, Egypt; 11 Egyptian Drug Authority (EDA), Cairo 11562, Egypt; mahfozamal@gmail.com; 12 Saudi Toxicology Society, Department of Pharmacology and Toxicology, College of Medicine, Umm Al Qura University, Makkah 21955, Saudi Arabia

**Keywords:** *Toxoplasma gondii*, hepatitis C virus, liver fibrosis, transient elastography, TIMP-1, M-CSF, YKL-40

## Abstract

Background: Chronic hepatitis C virus (HCV) infection remains an important cause of progressive liver disease and fibrosis. *Toxoplasma gondii* infection is highly prevalent worldwide and may influence inflammatory and fibrotic pathways; however, its clinical significance in patients with chronic HCV infection remains unclear. This study aimed to assess the prevalence of anti-*T. gondii* antibodies among patients with chronic HCV infection and to evaluate potential differences in liver fibrosis biomarkers and elastography findings according to *T. gondii* serostatus. Methods: In this case–control study conducted between December 2023 and October 2024 at Beni-Suef University Hospital, Beni-Suef, Egypt, 58 treatment-naive patients with chronic HCV infection, confirmed by documented anti-HCV positivity together with detectable serum HCV RNA, and 20 healthy controls were enrolled. Anti-*T. gondii* IgG and IgM antibodies were detected using a qualitative rapid immunochromatographic assay (OnSite Toxo IgG/IgM Combo Rapid Test, CTK Biotech, Poway, CA, USA). In HCV patients, liver fibrosis was assessed using transient elastography and circulating fibrosis-related biomarkers, including tissue inhibitor of metalloproteinase-1 (TIMP-1), macrophage colony-stimulating factor (M-CSF), and YKL-40, the latter three measured by quantitative sandwich enzyme-linked immunosorbent assay. Clinical, biochemical, and fibrosis-related parameters were compared between HCV patients and controls and according to *T. gondii* serological status. Results: Anti-*T. gondii* IgG seropositivity was detected in 26/58 (44.8%) patients with chronic HCV infection and 8/20 (40.0%) healthy controls, whereas IgM seropositivity was observed in 4/58 (6.9%) and 2/20 (10.0%), respectively. No statistically significant differences were observed between patients and controls in either IgG or IgM seropositivity rates. Patients with chronic HCV infection showed significantly higher circulating concentrations of TIMP-1, M-CSF, and YKL-40 than healthy controls (all *p* < 0.001). Among HCV patients, anti-*T. gondii* IgG serostatus was not significantly associated with FibroScan-defined fibrosis category, platelet count, AST, GGT, TIMP-1, M-CSF, or YKL-40. Analyses involving IgM-positive patients were considered exploratory because of the small number of IgM-positive participants. Conclusions: Anti-*T. gondii* IgG and IgM seropositivity did not differ significantly between patients with chronic HCV infection and healthy controls. Among HCV patients, serological evidence of previous *T. gondii* exposure was not significantly associated with transient elastography-defined fibrosis severity or the evaluated circulating fibrosis biomarkers. Because seropositivity reflects exposure rather than active parasitic infection, the findings should not be interpreted as evidence of active hepatic toxoplasmosis. Larger multicenter longitudinal studies incorporating molecular confirmation of active *T. gondii* infection are warranted.

## 1. Introduction

Hepatitis C virus (HCV) infection remains a major global health challenge and a leading cause of chronic liver disease, liver cirrhosis, and hepatocellular carcinoma [1,2,3]. Although the World Health Organization has established ambitious targets for HCV elimination by 2030, chronic HCV infection continues to impose a substantial public health burden, particularly in low- and middle-income countries, where the disease prevalence remains high [4]. The progression of HCV-related liver injury is influenced by multiple viral, host, metabolic, and environmental factors, highlighting the importance of identifying potential additional factors that may influence hepatic inflammation, tissue remodeling, and fibrosis progression [5].

*Toxoplasma gondii* is an obligate intracellular protozoan parasite that infects approximately one-third of the global population. Human infection is commonly acquired through the ingestion of tissue cysts in undercooked meat or oocysts that contaminate food, water, or soil. Although blood transfusion and organ transplantation have been reported as possible transmission routes, they are not considered primary modes of transmission [6]. In most immunocompetent individuals, infection remains asymptomatic and is characterized by persistent latent tissue cysts and long-lasting serological evidence of exposure. Accordingly, detection of anti-*T. gondii* IgG primarily reflects previous exposure, whereas IgM seropositivity may indicate more recent exposure but does not, by itself, establish active infection.

Interest has recently emerged regarding the potential interaction between *T. gondii* exposure and chronic liver diseases. Experimental studies, conducted largely in rodent models, together with observational clinical data, have suggested that *T. gondii* may induce hepatic inflammation, hepatocellular injury, and activation of immune pathways involved in tissue remodeling and fibrosis [1,7]. However, the available evidence regarding the relationship between *T. gondii* exposure and chronic HCV-associated liver fibrosis remains limited and inconsistent [8,9]. While some studies have reported higher seroprevalence rates among patients with chronic liver disease, others have failed to demonstrate significant associations between *T. gondii* seropositivity and fibrosis severity [10]. These discrepant findings indicate that the potential relationship between prior *T. gondii* exposure and the progression or severity of HCV-associated liver fibrosis remains uncertain.

The accurate assessment of liver fibrosis is essential for prognosis and the clinical management of patients with chronic HCV infection [11]. Although liver biopsy has traditionally been regarded as the reference standard for fibrosis evaluation, its invasive nature, sampling variability, and procedure-related complications have limited its widespread use. Consequently, non-invasive methods such as transient elastography and circulating fibrosis biomarkers have become increasingly important tools for fibrosis assessment [12]. However, fibrosis measurements may be influenced by concurrent inflammatory activity, metabolic abnormalities, and coexisting infections, potentially affecting their interpretation [13].

Among the circulating biomarkers associated with hepatic fibrogenesis, tissue inhibitor of metalloproteinase-1 (TIMP-1) [14], macrophage colony-stimulating factor (M-CSF) [15], and chitinase-3-like protein 1 (YKL-40) have shown potential value in reflecting fibrosis severity and extracellular matrix remodeling [16,17,18,19,20]. These biomarkers may therefore provide complementary information to transient elastography when evaluating fibrotic activity in patients with chronic liver disease. In addition, their reported links with immune and inflammatory responses associated with *T. gondii* infection provide a potential biological basis for investigating whether prior parasite exposure is related to fibrosis-associated pathways [21].

Despite these observations, data evaluating the relationship between *T. gondii* serological status and liver fibrosis in patients with chronic HCV infection remain limited, particularly when fibrosis is assessed using both transient elastography and circulating fibrosis-related biomarkers. Therefore, the present study aimed to determine the prevalence of anti-*T. gondii* IgG and IgM antibodies among patients with chronic HCV infection and to evaluate their association with liver fibrosis stage, transient elastography findings, biochemical liver parameters, and circulating fibrosis biomarkers.

## 2. Materials and Methods

### 2.1. Study Design and Population

This case–control study was conducted from December 2023 to October 2024 at the Internal Medicine Department and Hepatology Outpatient Clinics of Beni-Suef University Hospital, Beni-Suef, Egypt. The study was conducted as a single-center investigation.

A total of 78 participants were included, comprising 58 patients with chronic HCV infection and 20 apparently healthy controls. The HCV patients were recruited from the Hepatology Outpatient Clinics, while healthy controls were recruited from individuals attending the hospital who had no known history of chronic liver disease or HCV infection.

Inclusion criteria for the HCV group were: age 18 years or older; documented chronic HCV infection, defined in accordance with current European Association for the Study of the Liver recommendations as persistence of detectable serum HCV RNA for at least six months [22]; anti-HCV antibody positivity together with a positive HCV RNA polymerase chain reaction result recorded in the patient’s clinical file at or before enrolment; no previous interferon-based or direct-acting antiviral therapy, such that all enrolled patients were treatment-naive at the time of sampling; and provision of written informed consent.

Inclusion criteria for the control group were: age 18 years or older; absence of anti-HCV antibodies; no history or clinical evidence of chronic liver disease; and provision of written informed consent.

Exclusion criteria, applied to both groups, were: hepatitis B virus or human immunodeficiency virus co-infection; hepatocellular carcinoma or any other active malignancy; other established causes of chronic liver disease, including autoimmune hepatitis, primary biliary cholangitis, Wilson disease and hereditary haemochromatosis; hepatic schistosomiasis; significant alcohol consumption, defined as more than 30 g/day in men and more than 20 g/day in women; congestive hepatopathy; ongoing treatment with immunosuppressive agents or a known immunodeficiency state; pregnancy; and, in the HCV group, any condition precluding a reliable transient elastography examination, including ascites and a concurrent biochemical flare of hepatitis.

Because all patients were recruited from an established hepatology outpatient service, chronicity of infection was ascertained from documented anti-HCV positivity and HCV RNA detection recorded in the clinical file before the study period, rather than from a study-specific observation interval. As no patient had received antiviral therapy at the time of sampling, sustained virological response status was not applicable to this cohort.

The study protocol was reviewed and approved by the Faculty of Medicine, Beni-Suef University Research Ethics Committee (Approval No. FMBSUREC/03122023/Eid). The study was conducted in accordance with the ethical principles of the Declaration of Helsinki and its subsequent amendments. Written informed consent was obtained from all participants before enrollment and data collection.

All participant recruitment and study-related data collection were performed within the documented validity period of the ethics approval.

### 2.2. Clinical and Laboratory Assessment

All HCV patients underwent the following assessments:

- Detailed medical history taking;
- Physical examination;
- Body mass index (BMI) calculation;
- Complete blood count, including platelet count;
- Liver biochemical testing;
- Assessment of circulating fibrosis-related biomarkers (TIMP-1, M-CSF, and YKL-40);
- Serological evaluation for *Toxoplasma gondii* antibodies;
- Liver fibrosis assessment using transient elastography.

### 2.3. Assessment of HCV Infection

Serum samples were collected and centrifuged at 3000 rpm for 20 min. The separated sera were aliquoted and stored at −20 °C until analysis.

Anti-HCV antibodies and HCV core antigen were assessed using the Monolisa HCV Ag-Ab ULTRA V2 enzyme immunoassay (Bio-Rad Laboratories, Marnes-la-Coquette, France), according to the manufacturer’s instructions. This combined antigen–antibody assay was used for serological screening only and was not, by itself, taken as evidence of chronic infection or of active viraemia. In all 58 patients the diagnosis rested on a documented positive serum HCV RNA polymerase chain reaction result held in the clinical record. This follows current European Association for the Study of the Liver recommendations, under which detection of HCV RNA establishes current infection and its persistence beyond six months establishes chronic infection [22]. All 20 controls were negative for anti-HCV antibodies.

Participants were accordingly classified as treatment-naive chronic HCV cases or as healthy controls.

### 2.4. Assessment of Toxoplasma gondii Seropositivity

Anti-*Toxoplasma gondii* IgG and IgM antibodies were assessed using the OnSite Toxo IgG/IgM Combo Rapid Test (CTK Biotech Inc., Poway, CA, USA), according to the manufacturer’s instructions.

The assay is a qualitative lateral-flow immunochromatographic test. It reports anti-*T. gondii* IgG and IgM separately as present or absent, yields no antibody titre or quantitative index, and is therefore not an enzyme-linked immunosorbent assay. An independent Egyptian evaluation of this same commercial test, using ELISA as the reference standard, found a sensitivity of 74.0% and a specificity of 87.5% for anti-*T. gondii* antibodies, the two methods agreeing on 78.8% of 180 samples tested [23].

Samples producing invalid results were retested according to the manufacturer’s instructions.

For the present analysis, anti-*T. gondii* IgG and IgM seropositivity were considered markers of previous exposure or recent serological response, respectively, rather than definitive evidence of active infection. Because molecular confirmation was not performed, active *T. gondii* infection could not be independently confirmed.

### 2.5. Liver Fibrosis Assessment

Liver fibrosis was assessed in the HCV group using transient elastography (FibroScan 502 Touch, Echosens, Paris, France). Measurements were considered reliable when at least 10 valid measurements were obtained, the success rate exceeded 60%, and the interquartile range-to-median liver stiffness measurement ratio was 30% or less. The standard M probe was used routinely, whereas the XL probe was used in obese participants when required.

Patients were classified according to their liver stiffness measurement (LSM) as follows: F0–F1, no or minimal fibrosis (2–7 kPa); F2–F3, moderate to severe fibrosis (8–14 kPa); and F4, cirrhosis (>14 kPa).

Transient elastography was performed only in the HCV group and was used both to derive the continuous liver stiffness measurement and to assign each patient to one of these three fibrosis categories. Comparisons of fibrosis burden according to *T. gondii* serostatus were performed on the categorical fibrosis stage, as reported in Table 1.

### 2.6. Measurement of Fibrosis Biomarkers

Serum concentrations of the following fibrosis-related biomarkers were measured using commercially available quantitative sandwich ELISA kits according to the manufacturers’ protocols:

- Tissue inhibitor of metalloproteinase-1 (TIMP-1);
- Macrophage colony-stimulating factor (M-CSF); and
- Chitinase-3-like protein 1 (YKL-40).

All samples were analyzed in a single laboratory batch to minimize inter-assay variability and repeated freeze–thaw cycles.

### 2.7. Statistical Analysis

Statistical analyses were performed using IBM SPSS Statistics version 28.0 (IBM Corp., Armonk, NY, USA).

Continuous variables were tested for normality and presented as mean +/− standard deviation or median (interquartile range), as appropriate. Categorical variables were expressed as frequencies and percentages.

Comparisons between the 58 HCV patients and 20 healthy controls were performed using the independent-samples *t*-test or Mann–Whitney U test for continuous variables and the chi-square test or Fisher’s exact test for categorical variables, as appropriate.

Within the HCV group, clinical, biochemical, elastography, and fibrosis biomarker parameters were compared according to anti-*T. gondii* IgG and IgM serostatus. Because the number of IgM-positive HCV patients was small (n = 4), analyses according to IgM serostatus were considered exploratory and interpreted cautiously.

Correlation analyses were performed using Pearson’s or Spearman’s correlation coefficients according to data distribution.

A two-sided *p*-value < 0.05 was considered statistically significant.

Many clinical, biochemical, elastographic and biomarker parameters were compared according to anti-*T. gondii* serostatus, which inflates the type I error rate. The comparisons in Table 1 were therefore treated as two families of eight tests, one for IgG and one for IgM serostatus. Within each family, nominal *p*-values were adjusted by the Bonferroni method, giving a corrected threshold of alpha = 0.05/8 = 0.006, and by the Benjamini–Hochberg procedure controlling the false discovery rate at 5% [24]. Comparisons that lost significance after adjustment are treated as exploratory and hypothesis-generating rather than as established associations. Adjustment was confined to these secondary subgroup comparisons. The primary comparisons between HCV patients and controls, and the analyses of fibrosis biomarkers across fibrosis categories, were pre-specified and are reported unadjusted.

## 3. Results

### 3.1. Baseline Characteristics of the Study Population

A total of 78 participants were included in the final analysis, comprising 58 patients with chronic HCV infection and 20 healthy controls. The demographic and clinical characteristics of the study population, together with anti-*T. gondii* seropositivity rates, are presented in Table 2.

There were no statistically significant differences between HCV patients and healthy controls regarding age, sex distribution, or body mass index (BMI) (*p* > 0.05 for all comparisons). The mean age was 57.47 +/− 10.20 years among HCV patients and 54.45 +/− 5.96 years among controls (*p* = 0.116). Male participants comprised 51.7% of the HCV group and 45.0% of the control group (*p* = 0.604), while mean BMI was 28.45 +/− 4.11 and 29.26 +/− 2.01 kg/m^2^, respectively (*p* = 0.255).

Platelet counts were significantly lower in HCV patients than in healthy controls (181.89 +/− 99.34 vs. 252.05 +/− 49.93 × 10^3^/microL, respectively; *p* < 0.001).

Patients with chronic HCV infection also demonstrated significantly higher serum concentrations of TIMP-1, M-CSF, and YKL-40 compared with healthy controls (all *p* < 0.001), as well as higher gamma-glutamyl transferase and aspartate aminotransferase levels.

### 3.2. Anti-Toxoplasma gondii Seropositivity Among Study Participants

The prevalence of anti-*Toxoplasma gondii* antibodies among HCV patients and healthy controls is presented in Table 2.

Anti-*T. gondii* IgG seropositivity was detected in 26/58 (44.8%) HCV patients and 8/20 (40.0%) healthy controls. Anti-*T. gondii* IgM seropositivity was detected in 4/58 (6.9%) HCV patients and 2/20 (10.0%) healthy controls. No statistically significant differences were observed between HCV patients and controls regarding either IgG (*p* = 0.707) or IgM (*p* = 0.643) seropositivity.

Thus, the serological findings did not demonstrate a significant difference in *T. gondii* exposure between patients with chronic HCV infection and healthy controls.

### 3.3. Liver Fibrosis Parameters According to FibroScan Categories

Clinical, biochemical and biomarker parameters across transient elastography-defined fibrosis categories are presented in Table 3.

Among the 58 HCV patients, 26 (44.8%) were classified as having F0–F1 fibrosis, 10 (17.2%) as F2–F3, and 22 (37.9%) as F4 disease according to liver stiffness measurements.

Mean liver stiffness increased progressively across fibrosis categories, from 5.41 +/− 0.46 kPa in the F0–F1 group to 8.75 +/− 0.53 kPa in the F2–F3 group and 24.27 +/− 14.12 kPa in the F4 group (*p* < 0.001). Across the whole HCV cohort, the corresponding pooled liver stiffness measurement was 13.14 +/− 12.33 kPa, derived from the fibrosis-category data in Table 3.

BMI did not differ significantly across the three fibrosis categories (*p* = 0.160).

Patients with advanced fibrosis/cirrhosis demonstrated lower platelet counts and higher AST, GGT, TIMP-1, M-CSF, and YKL-40 concentrations compared with patients with less advanced fibrosis. The differences across fibrosis categories were statistically significant for these parameters.

### 3.4. Correlation of Fibrosis Biomarkers with Liver Stiffness and Laboratory Parameters

Correlations between the circulating fibrosis biomarkers, liver stiffness and laboratory parameters are presented in Table 4.

Serum TIMP-1, M-CSF, and YKL-40 concentrations showed strong positive correlations with liver stiffness measurements obtained by transient elastography (r = 0.832, 0.842, and 0.919, respectively; all *p* < 0.001).

In contrast, platelet counts showed significant inverse correlations with TIMP-1, M-CSF, and YKL-40 (r = −0.710, −0.804, and −0.761, respectively; all *p* < 0.001). Correlations between GGT and the fibrosis biomarkers were not statistically significant.

These findings indicate that the three evaluated circulating fibrosis biomarkers were strongly associated with liver stiffness and fibrosis severity in the HCV cohort.

### 3.5. Liver Fibrosis Parameters According to Anti-Toxoplasma gondii IgG Serostatus

Comparisons of clinical, laboratory, fibrosis biomarker, and elastography findings according to anti-*T. gondii* IgG serostatus are presented in Table 1.

Among the 58 HCV patients, 26 were anti-*T. gondii* IgG-positive and 32 were IgG-negative. Aspartate aminotransferase levels did not differ significantly according to IgG serostatus (32.88 +/− 19.50 versus 29.13 +/− 19.50 U/L; mean difference 3.75 U/L, 95% confidence interval −6.56 to 14.06; *p* = 0.470).

No statistically significant differences were observed between IgG-positive and IgG-negative patients regarding BMI, the distribution of transient elastography-based fibrosis categories, AST, platelet count, GGT, or the circulating fibrosis biomarkers TIMP-1, M-CSF, and YKL-40 (all *p* > 0.05).

After adjustment for multiplicity within this family of eight tests, no comparison by IgG serostatus remained statistically significant (all Bonferroni-adjusted *p* > 0.05; all Benjamini–Hochberg q > 0.05). The smallest nominal *p*-value was observed for GGT (*p* = 0.094), corresponding to a Benjamini–Hochberg q-value of 0.508.

Accordingly, anti-*T. gondii* IgG seropositivity was not significantly associated with fibrosis stage, biochemical markers of hepatic injury, or the evaluated circulating fibrosis biomarkers in this cohort.

No other comparison in either family remained significant after Bonferroni or Benjamini–Hochberg adjustment for eight tests per family.

### 3.6. Analysis According to Anti-Toxoplasma gondii IgM Serostatus

Comparisons according to anti-*T. gondii* IgM serostatus are also presented in Table 1.

Among the 58 HCV patients, 4 were anti-*T. gondii* IgM-positive and 54 were IgM-negative.

IgM-positive patients had a significantly lower mean BMI than IgM-negative patients (21.43 +/− 4.99 vs. 28.97 +/− 3.57 kg/m^2^, respectively; *p* < 0.001). However, because only four HCV patients were IgM-positive, this finding should be interpreted cautiously and considered exploratory. The difference persisted after adjustment for multiple testing (Bonferroni-adjusted *p* = 0.004) but was sensitive to the variance assumption: Welch’s unequal-variance *t*-test, which suits markedly unequal group sizes and variances, gave *p* = 0.054. The finding is therefore regarded as hypothesis-generating.

No statistically significant differences were observed between IgM-positive and IgM-negative patients regarding platelet count, AST, GGT, TIMP-1, M-CSF, or YKL-40 (all *p* > 0.05).

Similarly, no significant association was observed between IgM serostatus and the distribution of transient elastography-based fibrosis categories.

Given the very small number of IgM-positive participants, these subgroup findings should be regarded as exploratory rather than definitive.

## 4. Discussion

The present study investigated the association between *Toxoplasma gondii* seropositivity and liver fibrosis severity among Egyptian patients with chronic hepatitis C virus (HCV) infection using transient elastography and circulating fibrosis biomarkers [1,7].

Serum fibrosis biomarkers (YKL-40, TIMP-1, and M-CSF) were significantly associated with fibrosis severity, whereas no significant association was identified between *T. gondii* seropositivity and transient elastography-defined fibrosis categories or fibrosis biomarker levels. No significant association was observed between *T. gondii* serostatus and any biochemical marker of hepatic injury.

Patients with cirrhosis (F4) demonstrated significantly lower platelet counts, whereas AST, GGT, TIMP-1, M-CSF, and YKL-40 levels were highest in this group. These findings are consistent with progressive hepatic injury and fibrogenesis accompanying advanced liver disease.

Furthermore, YKL-40, TIMP-1, and M-CSF exhibited significant positive correlations with the FibroScan scores and AST levels and negative correlations with the platelet counts, supporting their role as indicators of hepatic fibrogenesis [17,18,19,25,26,27,28]. Haleem et al. (2015) speculated that YKL-40 with or without FibroScan can detect eligibility for antiviral treatment and monitor disease progression in patients undergoing therapy [29]. Moreover, Fontana et al. (2008) reported that YKL-40 and TIMP-1 in chronic HCV significantly correlate with the Ishak fibrosis stage [30]. Further, Preisser et al. demonstrated that patients with advanced fibrosis (F3–F4) showed higher serum levels of M-CSF compared to those with earlier stages (F0–F2), indicating that this cytokine is a profibrotic factor [18].

We noticed a significant inverse correlation between the fibrosis stage and platelet count. Previous work reported that the platelet count correlates negatively with fibrosis markers in HCV, with remarkable decreases in HCV-advanced fibrosis [14]. Bojanic et al. (2023) reported on noninvasive approaches to fibrosis assessment in chronic HCV infection [31]. Hence, the combination of conventional chemical and serological tests with FibroScan may be appropriate for proper liver fibrosis staging.

In the present study, the frequency of anti-*T. gondii* IgG seropositivity was slightly higher among HCV patients than healthy controls; however, the difference was not statistically significant. Likewise, anti-*T. gondii* IgM seropositivity was low and comparable between both groups. This finding is consistent with the results reported in a previous study in Mexico, where no significant difference (*p* = 0·610) was detected in Toxoplasma IgG seropositivity between cases of liver disease and their controls [32]. In contrast, our results conflict with those of previous studies from Egypt [33], Turkey [34], and China [35], which reported a significantly higher frequency of Toxoplasma IgG seropositivity among patients with chronic hepatitis, with prevalence rates of 76·9%, 68.5%, and 19·7%, respectively. Those studies suggested that chronic liver disease may be a potential risk factor for toxoplasmosis due to suppressed cell-mediated and humoral immune responses [33,34,35]. However, variations in the seroprevalence rates of *T. gondii* among different studies could be attributed to geographic differences, participant ages, diagnostic approaches used, or differences in the proportions of patients and controls [35].

Importantly, the present study assessed the serological evidence of exposure to *T. gondii* rather than active infection. The detection of anti-*T. gondii* IgG antibodies primarily reflects previous exposure and does not distinguish latent infection from active disease. Therefore, the findings should not be interpreted as evidence of active parasitic co-infection. Molecular methods such as polymerase chain reaction (PCR) would be required to confirm active infection.

The serological platform itself warrants comment. The assay used here is a qualitative immunochromatographic test rather than an ELISA, and against an ELISA reference standard in an independent Egyptian evaluation it showed a sensitivity of 74.0% and a specificity of 87.5% [23]. Sensitivity of that order means a proportion of previously exposed participants will have been classified as seronegative. The assay was applied identically to cases and controls and without knowledge of fibrosis stage, so any such misclassification would be non-differential, and non-differential misclassification of a dichotomous exposure attenuates measured associations towards the null. Our null findings must be read with that attenuation in mind. They are consistent with the absence of a meaningful association, but they cannot exclude a modest one that a more analytically sensitive assay might have detected. The same limitation may partly explain why our seroprevalence estimate falls below the rates reported from Egypt using ELISA-based platforms [33].

On the other hand, our results showed that the frequency of anti-*T. gondii* IgM antibodies did not differ significantly between the HCV patients and controls. This result is in concordance with various previous reports that observed a comparable seroprevalence of anti-*T. gondii* IgM antibodies between liver disease patients and controls [32,33,35].

Our findings revealed a relatively higher prevalence of *T. gondii* IgG positivity in fibrotic (F2-F3, 60.0%) and cirrhotic (F4, 45.5%) groups compared to the non-fibrotic group (38.5%), which might suggest increased past exposure to this parasitic infection among these patients. However, *T. gondii* IgG positivity did not differ significantly across the transient elastography-defined fibrosis categories, which may be due to the limited sample size.

A previous study reported serum Toxoplasma antigen (Tag) in 43.3% of cirrhotic cases versus 26.2% in fibrotic patients with chronic HCV infection [36]. Experimental work in mice also showed an increase in hepatic stellate cells in proportion to parasite antigen during acute infection [37]. Hepatic pathological changes, including necrosis, granuloma formation, hepatitis, and hepatomegaly, have also been described in experimental or clinical toxoplasmosis [38,39]. These reports derive from individual clinical cases and from population-level serological associations rather than from histological assessment of cohorts comparable to ours.

On the other hand, El-Nahas et al. (2014) did not find an association between liver fibrosis staging and Toxoplasma seroprevalence [33]. The inconsistent findings in previous studies regarding the association between the prevalence of *T. gondii* infection and liver fibrosis staging, together with the lack of statistical differences among different FibroScan scores in our results, suggest that this relationship remains unclear. This highlights the need for further research to comprehend this potential association.

The present study showed a nominally significant association between IgM seropositivity and low BMI, although this rested on only four IgM-positive patients and was not robust to the variance assumption used. A review by Dobner and Kaser (2018) showed that the risk of infection increases with being underweight [40]. Moreover, the current results did not show a significant association between BMI and IgG seroprevalence. Indeed, previous research has reported conflicting findings regarding the relationship between toxoplasmosis and obesity. For instance, Reeves et al. (2013) identified a positive association between *T. gondii* IgG seropositivity and obesity in Germany; however, the study provided no evidence of a causal relationship [41]. Another cross-sectional study from Germany reported that BMI ≥ 30 was an independent risk factor for *T. gondii* IgG seropositivity [42]. It has been hypothesized that weight gain associated with *T. gondii* infection may result from behavioral changes due to brain cysts leading to increased food intake or appetite, alteration in host lipid metabolism, or variations in *T. gondii* strains [41,43,44]. In contrast, a previous epidemiological study reported that *T. gondii* IgG seroprevalence and BMI were not associated [45]. Similarly, Alvarado-Esquivel et al. (2017) reported no serological (anti-IgM and anti-IgG antibodies) or molecular indication of an association between *T. gondii* infection and obesity [46]. The investigators attributed this discrepancy in the results of the link between *T. gondii* and BMI to differences in the study designs and the characteristics of the population studied [46].

The mechanistic evidence for hepatic involvement in *T. gondii* infection comes predominantly from experimental animal models. In mice inoculated with tachyzoites of a virulent strain, acute disseminated toxoplasmosis can involve the liver, spleen, and pancreas [47], and the number of hepatic stellate cells has been reported to increase in proportion to parasite antigen in liver tissue [37]. Altered hepatic trace-element composition has also been reported in infected rats [48], and DNA damage has been described in infected mice [36]. In humans, hepatic involvement has been documented mainly in isolated case reports of toxoplasmic hepatitis in immunocompetent individuals [39]. At the population level, a previous study reported that anti-*T. gondii* IgG seropositivity was associated with higher AST, ALT, GGT, and ALP and with an increased relative risk of chronic liver disease in a cross-sectional analysis of 3371 participants [38].

Two distinctions matter when these observations are placed beside our own. First, the animal work models acute, high-inoculum infection with a virulent laboratory strain, in one case with a uniformly fatal course [47], whereas our participants had serological evidence of remote, latent exposure in an immunocompetent state. These are different biological conditions rather than milder and more severe forms of the same one. Second, the human evidence, including that of a previous study [38], rests as ours does on antibody detection, and therefore describes a statistical association with previous exposure rather than a demonstrated hepatic process.

Our own study measured antibodies. No molecular testing for *T. gondii*, liver histology, immunohistochemistry or other assessment of parasite presence in hepatic tissue was undertaken, and it therefore offers no evidence either for or against hepatocyte invasion, active hepatic infection, or a direct parasitic contribution to liver injury in these patients. The experimental findings summarised above are cited as the biological rationale for the question we asked, not as processes demonstrated in this cohort.

A plausible reading of our own data is that, in patients with established and untreated chronic HCV infection, hepatic injury is dominated by the viral process to an extent that any additional contribution from remote *T. gondii* exposure is not detectable using routine aminotransferase measurement.

The serum fibrosis biomarkers did not show significant associations with anti-*T. gondii* IgG/IgM seropositivity. However, a study conducted by Shahad et al. (2022) reported that in acute and chronic Toxoplasma-seropositive patients, YKL-40 increased significantly compared to healthy controls [49]. The authors suggested that the YKL-40 biomarker may be useful to differentiate between the acute and chronic phases of Toxoplasma infection. Moreover, Tomasik et al. (2016) observed an increased expression of TIMP-1 along with other related biomarkers in the brains of *T. gondii*-infected mice, suggesting a possible role in the pathogenesis of cerebral toxoplasmosis [50].

An additional consideration is the timing of patient recruitment. Although Egypt has achieved remarkable success in reducing the burden of HCV through nationwide screening and treatment initiatives, including the “100 Million Healthy Lives” campaign, which screened approximately 57 million citizens and treated around four million between 2018 and 2019 [51], untreated chronic HCV patients continue to be encountered in tertiary referral centers because of delayed diagnosis, incomplete linkage to care, treatment deferral, or persistent active infection. Therefore, recruitment of untreated patients during the study period remained feasible despite the substantial progress of national HCV elimination programs.

### Strengths and Limitations

Several strengths should be acknowledged. First, the study evaluated *T. gondii* serological status together with objective non-invasive assessment of liver stiffness and multiple circulating fibrosis-related biomarkers. Second, inclusion of a healthy control group allowed comparison of *T. gondii* seroprevalence and fibrosis-related parameters between HCV patients and individuals without known chronic liver disease. Third, the study simultaneously examined serological, biochemical, elastographic, and biomarker-based measures of hepatic fibrosis. Fourth, chronic HCV infection was defined on the basis of documented HCV RNA positivity rather than serology alone, and all patients were treatment-naive, which removes the confounding effect of prior antiviral therapy on fibrosis measurements.

The study also has important limitations. First, its case–control design does not allow causal relationships to be established between *T. gondii* exposure and liver fibrosis. Second, the relatively small sample size limits the statistical power to detect modest associations, particularly in the IgM-positive subgroup, which included only four HCV patients. Third, *T. gondii* infection was assessed serologically, and molecular confirmation was not performed; therefore, the study cannot distinguish definitively between past exposure and active infection, and no assessment of the liver at tissue level, whether by histology, immunohistochemistry or molecular detection, was undertaken, so no inference about hepatic parasite involvement can be drawn from these data. Fourth, the study was conducted at a single center, which may limit the generalizability of the findings to other Egyptian populations or other geographic settings. Finally, potential residual confounding by demographic, environmental, metabolic, or clinical factors cannot be excluded.

Four further limitations arise from the methods. The serological assay was a qualitative immunochromatographic test rather than an ELISA, and its reported sensitivity of 74.0% against an ELISA reference standard [23] means some previously exposed participants will have been classified as seronegative. Such non-differential misclassification attenuates any true association towards the null, so our null findings indicate an absence of demonstrable association rather than positive evidence of no association. Because the assay is qualitative, antibody titres were unavailable and no dose–response relationship between the magnitude of the serological response and fibrosis burden could be examined. Comparisons of fibrosis burden by serostatus were made on categorical fibrosis stage rather than on continuous liver stiffness, which reduces statistical efficiency, and liver stiffness values are not tabulated for the control group, so no formal between-group comparison with controls is reported. Finally, the large number of subgroup comparisons is addressed by reporting Bonferroni- and Benjamini–Hochberg-adjusted values alongside nominal *p*-values [24]. After adjustment no comparison by IgG serostatus remained significant, and the one comparison that survived in the IgM family rested on four patients and was sensitive to the variance assumption. All subgroup findings should therefore be regarded as exploratory.

Taken together, these findings suggest that serological evidence of *T. gondii* exposure is relatively common among patients with chronic HCV infection but is not clearly associated with the severity of hepatic fibrosis as assessed by transient elastography or circulating fibrosis-related biomarkers. Nor was any association observed with biochemical markers of hepatic injury once multiplicity was taken into account.

## 5. Conclusions

In this single-center case–control study of 58 treatment-naive patients with chronic HCV infection and 20 healthy controls, anti-*Toxoplasma gondii* seropositivity was relatively common but was not significantly associated with transient elastography-defined fibrosis severity or circulating levels of TIMP-1, M-CSF, and YKL-40. No association was observed between anti-*T. gondii* serostatus and biochemical markers of hepatic injury once multiple testing was taken into account.

The very small number of IgM-positive HCV patients limits the interpretation of IgM subgroup analyses. Overall, the present findings do not support a clear association between serological evidence of *T. gondii* exposure and progression or severity of HCV-related liver fibrosis. Larger, multicenter, longitudinal studies incorporating more definitive assessment of active *T. gondii* infection and additional inflammatory and hepatic injury markers are warranted to clarify whether *T. gondii* exposure has any clinically relevant role in chronic HCV-associated liver disease.

## Figures and Tables

**Table 1:** Frequencies of anti-*T. gondii* IgG/IgM seropositivity in relation to the parameters and FibroScan scoring of HCV-positive patients (n = 58).

		*Toxoplasma* IgM			*Toxoplasma* IgG		
		Positive n = 4	Negative n= 54	*p* Value	Positive n = 26	Negative n = 32	*p* Value
**FibroScan scoring**	**F0–F1** **(n = 26)**	2 (7.7%)	24 (92.3%)	1	10(38.5%)	16 (61.5%)	0.538
	**F2–F3** **(n = 10)**	0	10 (100%)		6 (60%)	4(40%)	
	**F4** **(n= 22)**	2(9.1%)	20 (90.9%)		10 (45.5%)	12 (54.5%)	
**BMI** **(kg/m^2^)**		21.43± 4.99	28.97 ± 3.57	<0.001 *^b^	29.37 ± 4.61	27.71 ± 3.55	0.127
**PLT** **(10^3^/µL)**		245.25 ± 201.8	177.19 ± 89.35	0.918	178.4 ± 105.3	184.8 ± 95.86	0.579
**AST** **(U/L)**		23.75 ± 14.75	31.33 ± 19.8	0.486	32.88 ± 19.50	29.13 ± 19.5	0.470 ^a^
**GGT** **(U/L)**		46 ± 29.45	37.98 ± 20.6	0.779	33.37 ± 17.11	42.44 ± 23.4	0.094
**TIMP-1 ng/mL**		39.15 ± 18.01	49.10 ± 28.75	0.734	49.04 ± 26.95	47.9 ± 29.5	0.448
**M-CSF** **(ng/mL)**		0.58 ± 0.3	0.67 ± 0.50	0.871	0.68 ± 0.53	0.65 ± 0.46	0.516
**YKL-40** **(ng/mL)**		60.58 ± 27.5	77.85 ± 72.9	0.563	87.03 ± 98.46	68.23 ± 34.91	0.661

*: Statistically significant (*p* < 0.05). ^a^: Independent-samples *t*-test, t(56) = 0.73. Mean difference 3.75 U/L (95% confidence interval −6.56 to 14.06); standardised mean difference 0.19. ^b^: The IgM-positive versus IgM-negative BMI comparison remained significant after adjustment for multiple testing (Bonferroni-adjusted *p* = 0.004; Benjamini–Hochberg q = 0.004). It rests on four IgM-positive patients and is sensitive to the variance assumption: Welch’s unequal-variance *t*-test gives t(3.23) = −2.97, *p* = 0.054. It is therefore treated as exploratory.

**Table 2:** Parameters and characteristics of HCV patients and control groups: demographic data, serum fibrosis markers (YKL-40, TIMP-1, M-CSF), and Toxoplasma seropositivity.

		Control (n = 20)	Cases (n = 58)	*p* Value
**Age**	Mean ± SD	54.45 ± 5.96	57.47 ± 10.20	0.116
**Sex (No. %)**	Male	9 (45.0%)	30 (51.7%)	0.604
	Female	11(55.0%)	28 (48.3%)	
**BMI** **(kg/m^2^)**	Mean ± SD	29.26 ± 2.01	28.45 ± 4.11	0.255
**PLT** **(10^3^/µL)**	Mean ± SD	252.05 ± 49.93	181.89 ± 99.34	<0.001 *
**GGT** **(U/L)**	Mean ± SD	17.75 ± 5.76	38.37 ± 21.08	<0.001 *
**AST** **(U/L)**	Mean ± SD	19.60 ± 5.27	30.81 ± 19.46	0.01 *
**TIMP-1 (ng/mL)**	Mean ± SD	12.20 ± 4.97	48.41 ± 28.15	<0.001 *
**M-CSF** **(ng/mL)**	Mean ± SD	0.21± 0.04	0.66 ± 0.49	<0.001 *
**YKL-40** **(ng/mL)**	Mean ± SD	26.40 ± 6.96	76.66 ± 70.74	<0.001 *
***Toxoplasma*** **IgG** **NO. (%)**	Positive	8 (40.0%)	26 (44.8%)	0.707
	Negative	12 (60.0%)	32 (55.2%)	
***Toxoplasma*** **IgM** **NO. (%)**	Positive	2 (10.0%)	4 (6.9%)	0.643
	Negative	18 (90.0%)	54 (93.1%)	

*: Statistically significant (*p* < 0.05). TIMP-1: tissue inhibitor of metalloproteinases; YKL-40: chitinase-3-like protein; M-CSF: macrophage colony-stimulating factor. Transient elastography was not performed in the control group; therefore, liver stiffness measurements are not tabulated for controls and no formal between-group comparison of liver stiffness is reported.

**Table 3:** Parameters of HCV groups involving FibroScan scores, BMI, PLT, liver function tests, and serum fibrosis markers (YKL-40, TIMP-1, M-CSF) presented as the mean ± SD.

Parameter		F0–F1 (n = 26)	F2–F3 (n = 10)	F4 (n= 22)	*p* Value
BMI (kg/m^2^)	Mean ± SD	27.32 ± 4.81	29.72 ± 4.55	29.21 ± 2.53	0.160
LSM score (kPa)	Mean ± SD	5.41 ± 0.46 ^a,b^	8.75 ± 0.53 ^a,c^	24.27 ± 14.12 ^b,c^	<0.001 *
PLT (10^3^/µL)	Mean ± SD	251.08 ± 101.05 ^a^	184.70 ± 23.32 ^b^	98.84 ± 35.24 ^a,b^	<0.001 *
GGT (U/L)	Mean ± SD	33.69 ± 10.23	26.10 ± 11.49 ^a^	49.91 ± 28.06 ^a^	0.016 *
AST (U/L)	Mean ± SD	26.04 ± 19.91 ^a^	20.30 ± 5.93 ^b^	41.23 ± 18.61 ^a,b^	<0.001 *
TIMP-1 (ng/mL)	Mean ± SD	29.46 ± 8.62 ^a,b^	43.14 ± 3.77 ^a^	73.21 ± 30.88 ^b^	<0.001 *
M-CSF (ng/mL)	Mean ± SD	0.37 ± 0.09 ^a,b^	0.55 ± 0.07 ^a^	1.06 ± 0.61 ^b^	<0.001 *
YKL-40 (ng/mL)	Mean ± SD	40.74 ± 6.88 ^a,b^	64.67 ± 10.07 ^a^	124.55 ± 97.02 ^b^	<0.001 *

*: Statistically significant (*p* < 0.05), using the Kruskal–Wallis test. ^a,b,c^: These denote a significant difference (*p* < 0.05) between any two groups, within the same row, having the same superscript letter, using post hoc pairwise comparisons. BMI: body mass index; GGT: gamma-glutamyl transferase; AST: aspartate aminotransferase; PLT: platelets; TIMP-1: tissue inhibitor of metalloproteinases; YKL-40: chitinase-3-like protein; M-CSF: macrophage colony-stimulating factor.

**Table 4:** Correlation of serum fibrosis markers with liver stiffness measurements and the liver function parameters in HCV cases (n = 58).

		TIMP-1	M-CSF	YKL-40
**LSM score**	R	0.832	0.842	0.919
	*p* value	<0.001 *	<0.001 *	<0.001 *
**PLT**	R	−0.710	−0.804	−0.761
	*p* value	<0.001 *	<0.001 *	<0.001 *
**GGT**	R	0.133	0.205	0.205
	*p* value	0.321	0.123	0.123
**AST**	R	0.379	0.474	0.386
	*p* value	0.003 *	<0.001 *	0.003 *

*: Statistically significant (*p* < 0.05). R: correlation coefficient; LSM: liver stiffness measurements (FibroScan score); GGT: gamma-glutamyl transferase; AST: aspartate aminotransferase; PLT: platelets; TIMP-1: tissue inhibitor of metalloproteinases; YKL-40: chitinase-3-like protein; M-CSF: macrophage colony-stimulating factor.

## Data Availability

Raw data supporting this study’s findings are available from the corresponding author upon reasonable request.
